# Unsupervised Clinical Phenotyping Identifies Distinct Risk Profiles in Incisional Hernia Repair

**DOI:** 10.3390/medicina62061193

**Published:** 2026-06-21

**Authors:** Laurențiu Augustus Barbu, Daniel Ioan Mihalache, Liviu Vasile, Stelian-Stefaniță Mogoantă, Tiberiu Stefăniță Țenea Cojan, Nicolae-Dragoș Mărgăritescu, Gabriel Florin Răzvan Mogoș

**Affiliations:** 1 Department of Surgery, Railway Clinical Hospital Craiova, University of Medicine and Pharmacy of Craiova, 2 Petru Rares Street, 200349 Craiova, Romania; laurentiu.barbu@umfcv.ro (L.A.B.); tiberiu.tenea@umfcv.ro (T.S.Ț.C.); gabriel.mogos@umfcv.ro (G.F.R.M.); 2 Doctoral School, “Carol Davila” University of Medicine and Pharmacy, 37 Dionisie Lupu Street, 020021 Bucharest, Romania; 3 Department of Surgery, Emergency County Hospital, University of Medicine and Pharmacy of Craiova, 2 Petru Rares Street, 200349 Craiova, Romania; ssmogo@yahoo.com (S.-S.M.); dmargaritescu@yahoo.com (N.-D.M.)

**Keywords:** incisional hernia, abdominal wall reconstruction, clinical phenotyping, risk stratification, precision surgery, hernia recurrence

## Abstract

*Background and Objectives*: Patients undergoing incisional hernia repair constitute a clinically heterogeneous population with variable postoperative outcomes. Conventional risk models based on isolated risk factors may inadequately capture this complexity. This study aimed to identify data-driven clinical phenotypes and evaluate their association with surgical outcomes. *Methods and Materials*: A retrospective cohort of 1262 patients undergoing retromuscular incisional hernia repair (Rives–Stoppa technique) was analyzed. Unsupervised clinical phenotyping was performed using latent class analysis based on seven preoperative variables. Model selection was guided by Akaike information criterion (AIC), Bayesian information criterion (BIC), entropy, and clinical interpretability. Postoperative outcomes were compared across phenotypes. *Results*: Three distinct phenotypes were identified: metabolic (34.6%), structural (33.9%), and frailty (31.5%). The structural phenotype showed the highest complication (22.7%) and recurrence rates (8.6%), while the frailty phenotype had the lowest complication burden (14.6%). The metabolic phenotype was characterized by obesity and diabetes, consistent with increased wound-related morbidity. Cluster robustness was supported by internal validation metrics and sensitivity analyses. *Conclusions*: In this retrospective single-center cohort, distinct clinical phenotypes with different outcome profiles were identified among patients undergoing incisional hernia repair, supporting the concept that this population comprises clinically heterogeneous subgroups with distinct patterns of vulnerability. These findings should be considered preliminary and hypothesis-generating. Further external validation and prospective studies are required to determine the clinical utility of phenotype-based risk stratification.

## 1. Introduction

Incisional hernia remains a major challenge in abdominal wall surgery, associated with substantial morbidity, impaired quality of life, frequent reoperation, and significant healthcare costs. Current European Hernia Society (EHS) guidelines recognize incisional hernia as a complex and heterogeneous condition in which patient-related factors, defect characteristics, and operative variables interact to influence postoperative outcomes [1]. Despite ongoing advances in abdominal wall reconstruction, mesh technology, and perioperative optimization, substantial variability in outcomes persists among patients classified as high risk, suggesting considerable biological and clinical heterogeneity within this population [1,2]. Moreover, contemporary evidence supports the interaction between patient biology, fascial biomechanics, and reconstructive factors in determining postoperative outcomes [2,3].

Traditional risk assessment in incisional hernia repair has largely relied on isolated predictors such as obesity, smoking, diabetes, frailty, defect size, recurrence, and operative complexity. Although these factors are associated with complications and recurrence, conventional risk models assume additive effects and may inadequately capture multidimensional interactions among coexisting vulnerabilities. Existing models have shown only moderate discrimination, limited transportability across populations, and may oversimplify the heterogeneity encompassed within the broad category of “high-risk” patients [4,5]. In addition, obesity may demonstrate threshold-dependent rather than purely binary effects on morbidity, further suggesting that risk may be better understood as multidimensional rather than factor-specific [6].

Increasingly, evidence from other heterogeneous diseases suggests that complex patient populations may be better characterized as distinct phenotypic subgroups rather than homogeneous risk strata. Unsupervised clustering approaches have identified clinically meaningful phenotypes in conditions such as heart failure, sepsis, and acute respiratory distress syndrome, supporting the concept that data-driven subgroups may reveal latent clinical structure not captured by traditional models [7,8,9].

In abdominal wall reconstruction, however, phenotype-based stratification remains minimally explored. Incisional hernia patients frequently exhibit overlapping metabolic, structural, and physiologic vulnerabilities that may cluster into distinct patterns associated with different mechanisms of failure. Such heterogeneity raises the possibility that “high-risk” does not represent a single clinical entity, but rather multiple biologically and surgically relevant subgroups. This may be particularly relevant in retromuscular reconstruction, where outcomes reflect a complex interaction between patient factors, defect mechanics, and reconstructive strategy. Furthermore, emerging evidence suggests that patients undergoing incisional hernia repair may not benefit equally from uniform preventive or reconstructive approaches, supporting the need to identify clinically meaningful clinical subgroups [1].

Data-driven phenotyping may offer a complementary framework to conventional risk prediction by identifying latent clinical subgroups that may reflect distinct mechanisms of vulnerability. Such an approach may improve biologic interpretability, refine risk stratification, and support precision surgery strategies in which prehabilitation, operative planning, and perioperative optimization are adapted according to phenotype-specific vulnerabilities [3,5,6]. Given the growing availability of structured perioperative data and advances in unsupervised machine learning, such approaches are increasingly feasible in surgical populations [5,9]. However, given the limited prior application of phenotyping in abdominal wall reconstruction, this approach should presently be considered hypothesis-generating and requiring future external validation.

We therefore hypothesized that patients undergoing retromuscular incisional hernia repair represent a heterogeneous population comprising distinct clinical phenotypes identifiable through unsupervised cluster analysis, and that these phenotypes are associated with differential postoperative outcomes and clinically relevant patterns of vulnerability. The aim of this study was to identify and internally validate phenotype clusters in a large cohort of patients undergoing retromuscular incisional hernia repair and to explore their potential implications for precision risk stratification and personalized abdominal wall reconstruction.

## 2. Materials and Methods

### 2.1. Study Design and Population

This retrospective cohort study included 1262 consecutive patients who underwent retromuscular incisional hernia repair using the Rives–Stoppa technique at the Emergency County Hospital of Ploiești, Romania, between January 2013 and December 2022. This cohort has been described previously in a study evaluating predictors of postoperative complications using conventional regression-based methods. The present investigation represents a separate secondary analysis of the same dataset and was designed to address a different research objective, namely, the identification of latent clinical phenotypes through unsupervised clustering and the exploration of patient heterogeneity beyond individual risk-factor assessment.

### 2.2. Ethical Approval

The study protocol was reviewed and approved by the Ethics Committee of the Emergency County Hospital of Ploiești. Ethical approval was granted following evaluation of the research proposal submitted to the institutional review board (approval no. 13741/24 March 2023).

Given the retrospective nature of the study, the requirement for informed consent was waived in accordance with institutional policies.

### 2.3. Data Collection and Variables

Clinical and perioperative data were extracted from institutional medical records. Preoperative variables included age, body mass index (BMI), diabetes mellitus, smoking status, defect size, recurrence status, and American Society of Anesthesiologists (ASA) classification.

These variables were selected based on their established clinical relevance in incisional hernia outcomes and were used for phenotype construction. The objective was not to provide an exhaustive representation of patient complexity, but rather to evaluate whether a parsimonious set of well-established preoperative risk factors could identify clinically meaningful latent subgroups. Variables were selected a priori based on consistent evidence linking them to postoperative morbidity, recurrence, and perioperative risk in incisional hernia repair.

Missing data were minimal (<5% for all variables) and were handled using complete-case analysis.

### 2.4. Phenotype Identification

Unsupervised clinical phenotyping was performed using latent class analysis (LCA) based on seven preoperative categorical variables: age > 65 years, obesity (BMI ≥ 30 kg/m^2^), diabetes mellitus, active/recent smoking, large defect (W3), recurrent hernia, and ASA III–IV status. Continuous variables were categorized using clinically established thresholds commonly applied in abdominal wall reconstruction and perioperative risk assessment (age > 65 years, BMI ≥ 30 kg/m^2^, and ASA III–IV). This approach was selected to improve clinical interpretability of latent classes and to facilitate identification of phenotypes based on readily applicable risk characteristics. The objective was to derive clinically interpretable phenotypes rather than maximize predictive performance. We acknowledge that categorization may reduce information granularity; therefore, the resulting phenotypes should be interpreted as pragmatic clinical constructs. We acknowledge that categorization may reduce information granularity; therefore, sensitivity analyses were performed to evaluate phenotype stability under alternative model specifications.

Latent class models were estimated using XLSTAT 2022 (Addinsoft SARL, Paris, France). Models with two to four latent classes were evaluated. The optimal number of classes was selected according to Akaike information criterion (AIC), Bayesian information criterion (BIC), entropy, and clinical interpretability.

Each patient was assigned to the phenotype for which the posterior membership probability was highest.

Cluster robustness and separation were further evaluated using internal validation metrics, including silhouette coefficient, Davies–Bouldin index, and Calinski–Harabasz index, as well as sensitivity analyses under alternative model specifications.

### 2.5. Follow-Up and Outcome Assessment

Postoperative outcomes included seroma, surgical site infection (SSI), hematoma, hernia recurrence, and overall complications. Outcome assessment was based on documentation recorded during postoperative outpatient visits and review of hospital records. Seroma, SSI, and hematoma were identified according to the treating surgeon’s clinical diagnosis as documented in the medical records. Hernia recurrence was defined as a clinically or radiologically documented recurrent abdominal wall defect at the repair site. Overall complications were defined as the occurrence of any recorded postoperative complication. Because of the retrospective study design, outcomes were identified from routine clinical documentation rather than a prospectively standardized adjudication protocol.

### 2.6. Statistical Analysis

All statistical tests were two-tailed, and a *p*-value < 0.05 was considered statistically significant.

Data preprocessing was performed using Microsoft Excel 2016 (Microsoft Corp., Redmond, WA, USA) with XLSTAT 2022 (Addinsoft SARL, Paris, France). Statistical analyses were conducted using SPSS version 27.0 (IBM Corp., Armonk, NY, USA).

Categorical variables were compared using the chi-square test, and continuous variables were analyzed using appropriate parametric or non-parametric tests, as applicable. Multinomial logistic regression analysis was performed to identify independent predictors of phenotype membership.

## 3. Results

Among 1262 patients, obesity was the most prevalent risk feature (52.0%), followed by age > 65 years (36.0%) and ASA III–IV status (35.0%). Recurrent hernia was present in 23.0% of patients, while diabetes, smoking, and large defects (W3) were each identified in approximately 20% of cases (Table 1). These findings supported significant clinical heterogeneity within the cohort for phenotype analysis.

Model fit analysis supported a three-cluster solution as the optimal phenotype structure, showing the lowest BIC and highest entropy compared with the two- and four-cluster models (Table 2).

Cluster analysis identified three distinct clinical phenotypes with relatively balanced distribution: metabolic phenotype (34.6%), structural phenotype (33.9%), and frailty phenotype (31.5%) (Table 3).

Baseline characteristics differed significantly across phenotypes. The metabolic phenotype was dominated by obesity and diabetes, the structural phenotype by large defects and recurrent hernia, while the frailty phenotype showed the highest prevalence of advanced age and ASA III–IV status (all *p* < 0.05) (Table 4).

Postoperative outcomes differed across phenotypes, with the structural phenotype showing the highest overall complication rate, while the frailty phenotype had the lowest. Significant differences were observed for overall complications and seroma formation (Table 5).

Clinical interpretation showed that each phenotype was associated with a distinct biological profile, specific surgical vulnerability, and potentially different optimization strategy (Table 6).

Internal validation metrics confirmed that the three-cluster solution showed the best separation and robustness compared with the two- and four-cluster models (Table 7).

Sensitivity analyses demonstrated stable phenotype distribution across alternative model specifications, supporting the robustness of the identified cluster structure (Table 8).

Post hoc analysis showed significantly higher recurrence in the structural phenotype compared with the frailty phenotype, while no significant difference was observed between metabolic and frailty phenotypes (Table 9).

Multinomial regression identified large defect (W3), recurrent hernia, and ASA III–IV as independent predictors of membership in the structural phenotype, while obesity was inversely associated (Table 10).

**Figure 1:**
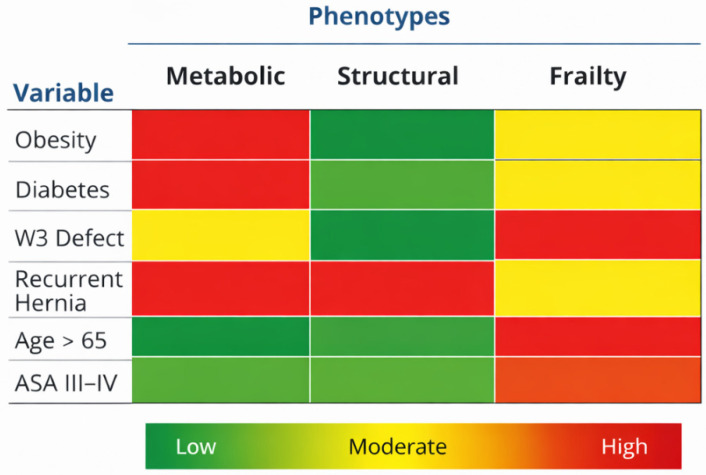
Heatmap of Clinical Variable Enrichment Across Phenotype Clusters.

Distinct enrichment patterns of clinical variables across phenotype clusters were observed, as shown in Figure 1.

**Figure 2:**
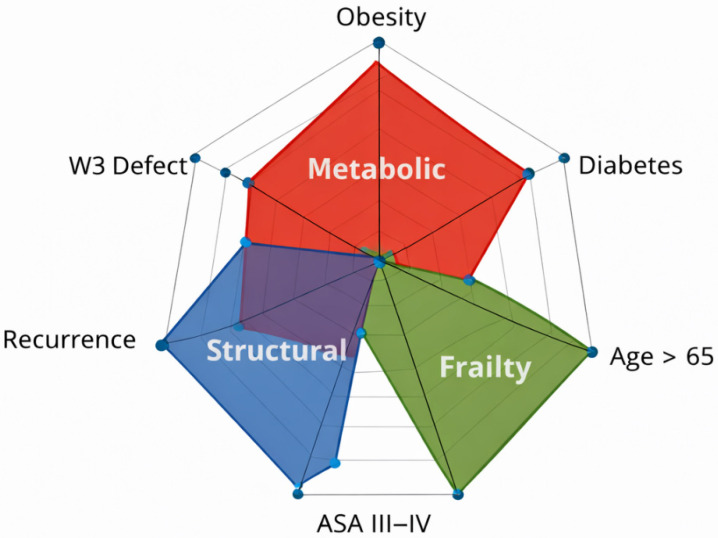
Radar Plot of Multidimensional Profiles Across Clinical Phenotype Clusters.

Distinct multidimensional profiles of the identified phenotypes are visualized in Figure 2.

## 4. Discussion

### 4.1. Principal Findings

In this study of 1262 consecutive patients undergoing retromuscular incisional hernia repair, we identified three clinically distinct phenotypes—metabolic, structural, and frailty—using an unsupervised clustering approach. These findings indicate that patients commonly grouped under the broad label of “high-risk” do not represent a homogeneous surgical population, but rather comprise biologically and clinically different subgroups with distinct patterns of vulnerability. This observation is consistent with the broader concept that complex clinical syndromes are often heterogeneous and may be better understood through data-driven subgroup identification rather than conventional one-size-fits-all classification systems [10,11,12].

A second major finding is that these phenotypes were not merely descriptive but were associated with differential postoperative outcomes. The structural phenotype showed the highest overall complication burden and the highest recurrence rate, whereas the frailty phenotype, despite advanced age and a markedly elevated prevalence of ASA III–IV status, demonstrated lower complication and recurrence rates than might be expected. This comparatively favorable outcome profile deserves careful interpretation.

Although the frailty phenotype was characterized by advanced age and a high prevalence of ASA III–IV status, it demonstrated substantially lower rates of large defects and recurrent hernias than the structural phenotype. This suggests that postoperative morbidity and recurrence in retromuscular incisional hernia repair may be influenced not only by physiologic vulnerability but also by the underlying mechanical complexity of the abdominal wall defect. Consequently, structural disease burden may have outweighed systemic frailty as a determinant of surgical failure in the present cohort.

Alternative explanations should also be considered. Because this was a retrospective surgical cohort, selection bias cannot be excluded. Frail patients who ultimately underwent elective reconstruction may have represented a relatively optimized subgroup after preoperative assessment and risk selection, whereas more severely vulnerable patients may not have been offered surgery. Residual confounding related to unmeasured clinical factors may also have contributed to the observed outcome differences. These findings should therefore not be interpreted as suggesting that frailty is protective, but rather that different dimensions of risk may contribute differently to postoperative outcomes in abdominal wall reconstruction.

The metabolic phenotype, in turn, was characterized by marked enrichment in obesity and diabetes, supporting a biologically plausible link with impaired healing and wound-related morbidity. This is clinically important because it suggests that risk may not be fully explained by isolated variables alone, but by how these variables cluster within patients. Similar phenotype-based differences in prognosis and treatment relevance have been reported in heart failure, ARDS, and sepsis, where cluster membership captured clinically meaningful patterns beyond single predictors [10,11,13].

Third, the three-cluster solution showed the most favorable combination of statistical fit, separation, and interpretability. In particular, the internal validation profile of the selected model was strengthened by entropy, silhouette coefficient, Davies–Bouldin index, Calinski–Harabasz index, and stability across sensitivity analyses. This is important because one of the most common criticisms of cluster-based studies is that subgroup structure may be unstable or data-dependent. In the present study, however, the convergence of multiple internal metrics supports the robustness of the identified phenotype structure. Similar emphasis on internal validation and stability assessment has been considered essential in phenotype research across other heterogeneous medical conditions [10,12,13].

### 4.2. Interpretation in the Context of Existing Literature

Current risk assessment in abdominal wall reconstruction remains largely based on isolated risk factors and traditional regression-based tools. Obesity, smoking, diabetes, frailty, defect size, and recurrence are all established contributors to postoperative morbidity, but existing models may incompletely represent the multidimensional interaction between biologic reserve, wound healing, and structural complexity [14,15,16]. This issue may be amplified by the broader evidence base in ventral and incisional hernia surgery, which remains limited by substantial heterogeneity in techniques, outcome definitions, and follow-up, thereby complicating the development of universally generalizable conventional risk models [14,17].

Current European Hernia Society guidelines emphasize that outcomes after incisional hernia repair are influenced by the interaction between patient-related risk factors, defect characteristics, and technical aspects of reconstruction [1]. The phenotype structure identified in the present study is broadly consistent with this multidimensional framework, although it was derived through an unsupervised analytical approach rather than predefined clinical classification.

In this context, the present study builds on prior regression-based analyses in retromuscular incisional hernia repair by moving beyond isolated predictors of complications toward multidimensional and clinically interpretable patient subgroups [18].

Importantly, the present study addresses a different research question from our previously published regression-based analysis of the same surgical cohort [19]. The earlier study sought to identify independent predictors of postoperative complications using supervised statistical modeling, focusing on the individual contribution of specific risk factors. In contrast, the current investigation uses an unsupervised latent class approach to examine whether these risk factors cluster within patients into distinct multidimensional phenotypes. Rather than estimating the effect size of individual predictors, the objective of the present study was to explore latent subgroup structure and determine whether clinically meaningful patient phenotypes could be identified.

Notably, the phenotypes identified in the present analysis were broadly consistent with the risk domains highlighted in the previous regression-based study. Importantly, the previous regression-based analysis provided greater granularity regarding the independent contribution of individual risk factors to postoperative complications. In contrast, the current study sacrifices some variable-level detail in order to evaluate whether these factors co-occur within clinically meaningful patient subgroups. Thus, the two approaches provide complementary perspectives: one focused on quantifying the effect of individual predictors, and the other focused on characterizing multidimensional patterns of patient vulnerability.

Variables previously associated with postoperative complications, including obesity, ASA status, recurrent hernia, and defect complexity, emerged as key components of the metabolic, frailty, and structural phenotypes. However, whereas the prior analysis evaluated these factors individually, the current approach suggests that they cluster within patients into distinct patterns of vulnerability that may not be fully captured by isolated risk-factor assessment alone.

Thus, the two analyses should be viewed as complementary rather than redundant, as they address different methodological and clinical questions. The previous study focused on identifying predictors of adverse outcomes, whereas the current study focused on identifying latent patient subgroups that may provide a broader framework for understanding heterogeneity among patients undergoing retromuscular incisional hernia repair.

Our findings extend this literature by suggesting that these variables may organize into latent clinical phenotypes with different mechanisms of failure. The metabolic phenotype appears to capture a wound-healing axis dominated by obesity and diabetes. This interpretation is supported by prior literature linking obesity to greater morbidity in ventral hernia repair and by evidence that wound complications share overlapping biological pathways with incisional hernia development [6,16,20,21]. This interpretation is also supported by contemporary data suggesting that obesity may exert threshold-dependent rather than purely binary effects on postoperative morbidity in ventral hernia repair, reinforcing the concept that metabolic risk may be better represented as part of a broader vulnerability phenotype than as an isolated variable alone [6]. Prior work also suggests that infectious complications should not be viewed in isolation, as deep and complex infections may involve multiple interconnected pathophysiological pathways and anatomical planes, contributing to more severe clinical trajectories [2,16,21,22].

The biological plausibility of these phenotype-specific trajectories is reinforced by evidence that chronic implant-related inflammation, fibrosis, and tissue remodeling may substantially influence abdominal wall healing and prosthesis–host tissue integration [23].

The structural phenotype appears to capture a mechanical failure axis dominated by large defects and recurrent hernia. This is highly plausible from an abdominal wall reconstruction perspective, as large defects, loss of domain, and recurrent hernias are consistently associated with technical complexity and recurrence risk in ventral hernia surgery, findings that are also recognized in European Hernia Society guidance regarding abdominal wall reconstruction and closure strategies [1,2,14,24,25]. This interpretation is further supported by the contemporary literature showing that abdominal wall outcomes are strongly influenced by fascial biomechanics, closure strategy, and the interaction between tissue quality and mechanical load, reinforcing the concept that structural failure risk is not merely anatomical, but biomechanical [2,18,26,27,28].

The frailty phenotype was characterized predominantly by age and ASA III–IV status, representing a systemic physiologic vulnerability axis. Importantly, frailty in hernia populations may not translate uniformly into worse patient-centered or recurrence outcomes, which supports the concept that frailty represents a distinct dimension of vulnerability rather than a simple proxy for technical failure risk [27,28,29]. Newly available hernia-specific patient-reported outcome data further support this interpretation, showing that although frail patients are typically older, more comorbid, and may present with larger hernias, frailty status was not independently associated with postoperative decision regret after elective ventral and incisional hernia repair, suggesting that clinical vulnerability and patient-perceived benefit may not always align [29].

A likely reviewer concern is that the phenotypes may partly reflect variables already chosen by the investigators. This criticism deserves acknowledgment. However, although the input variables were clinically selected, phenotype assignment itself emerged through an unsupervised clustering process and was supported by internal validation metrics and stability analyses, suggesting non-random latent structure rather than purely investigator-imposed categorization. In this sense, the present work is aligned with other phenotype studies in heterogeneous diseases, where clinically selected variables still yielded emergent subgroup structure with prognostic and therapeutic relevance [10,11,13,30,31,32].

### 4.3. Clinical Implications

The potential clinical relevance of these findings is that phenotype-based stratification may offer a complementary framework for understanding clinical heterogeneity among patients undergoing retromuscular incisional hernia repair beyond conventional risk-factor assessment alone. The identified phenotypes were associated with distinct patterns of vulnerability, suggesting that different patient subgroups may warrant further investigation in future studies evaluating tailored perioperative approaches.

The metabolic phenotype was characterized predominantly by obesity and diabetes, factors that are already recognized in current European Hernia Society guidance and contemporary abdominal wall reconstruction literature as important targets for preoperative optimization [1,14,33]. Rather than introducing new management principles, the present findings suggest that phenotype-based classification may provide an additional framework for identifying patients in whom these established optimization strategies are particularly relevant. Whether phenotype-guided prioritization of such interventions improves outcomes remains unknown and requires prospective evaluation.

The structural phenotype was characterized by large defects and recurrent hernias, features commonly associated with technical complexity and recurrence risk [1,2,14,34]. Similarly, the reconstruction-focused considerations associated with this phenotype are broadly consistent with existing guideline-based principles emphasizing defect complexity, recurrence risk, and technical planning in abdominal wall reconstruction [1]. The potential contribution of phenotyping is therefore not the introduction of new reconstructive strategies, but the possibility of identifying patient subgroups in whom these established approaches may be especially important.

The frailty phenotype was characterized primarily by advanced age and elevated ASA III–IV status, suggesting a predominance of systemic physiologic vulnerability. This phenotype may therefore identify patients in whom perioperative optimization, geriatric assessment, shared decision-making, and physiologic risk-reduction strategies could be explored prospectively. At the same time, recent hernia-specific evidence suggests that frailty should not automatically be equated with poor patient-perceived operative value, as frail patients may not experience greater postoperative decision regret despite increased baseline vulnerability [29,35].

More broadly, these observations are consistent with the growing interest in phenotype-oriented approaches across heterogeneous medical conditions [11,29]. However, unlike established therapeutic strategies, phenotype-guided management has not been evaluated in the present study. Therefore, any proposed phenotype-specific interventions should be regarded as hypotheses for future investigation rather than evidence-based clinical recommendations. As highlighted in other phenotype-guided fields, the clinical utility of such stratification ultimately depends on whether subtype-informed management improves outcomes prospectively, which remains to be demonstrated in abdominal wall reconstruction [11,36].

In addition, recent work in abdominal wall reconstruction has highlighted the growing potential of imaging-based artificial intelligence tools to support preoperative planning and complication prediction, suggesting that future phenotyping frameworks may integrate both clinical and imaging-derived data for more precise risk characterization [4,37].

Thus, the present study does not simply show that clusters exist; it suggests that clinically distinct subgroups of patients undergoing retromuscular incisional hernia repair may exhibit different patterns of vulnerability that could potentially be relevant for perioperative planning and risk assessment. However, the clinical utility of phenotype-based stratification remains hypothetical and requires prospective evaluation to determine whether phenotype-informed management improves patient outcomes.

Importantly, the phenotype-specific considerations discussed above should not be interpreted as novel treatment recommendations. Most are already reflected in contemporary abdominal wall reconstruction guidelines, including European Hernia Society guidance [1]. The potential value of phenotyping lies in providing an alternative framework for patient stratification and hypothesis generation, rather than establishing new standards of care.

### 4.4. Strengths and Limitations

This study has several strengths. First, to our knowledge, it is among the first to apply unsupervised clinical phenotyping specifically to high-risk incisional hernia repair. Second, the study included a relatively large cohort of 1262 patients, allowing identification of balanced phenotypic groups with sufficient clinical interpretability. Third, the phenotypes were supported by several complementary internal validation metrics and sensitivity analyses, strengthening confidence in cluster robustness. Fourth, the phenotypes were clinically coherent and linked to actionable implications, which improves the translational relevance of the findings. Finally, the study moves beyond conventional single-variable risk analysis toward a phenotype-based framework that may better reflect the true heterogeneity of complex abdominal wall reconstruction [10,11,13].

Several limitations should also be acknowledged. First, this was a retrospective single-center study, which may introduce selection bias and limit external generalizability. In addition, all patients underwent retromuscular incisional hernia repair using the Rives–Stoppa technique. Although this improved procedural homogeneity and reduced variability related to surgical approach, the identified phenotypes may be influenced by patient selection and technical factors specific to open retromuscular reconstruction. Consequently, their applicability to minimally invasive repairs, component separation techniques, bridged reconstructions, or alternative mesh placement strategies remains uncertain. Second, although the phenotypes emerged through unsupervised clustering and demonstrated good internal robustness, external validation in independent multicenter cohorts and across diverse abdominal wall reconstruction approaches is necessary before broader clinical application. Furthermore, the study population was derived from a single Romanian tertiary referral center and was therefore relatively geographically and ethnically homogeneous. Differences in demographic characteristics, genetic background, socioeconomic factors, healthcare systems, referral patterns, and comorbidity profiles across populations may influence phenotype structure and distribution. Consequently, the reproducibility and generalizability of the identified phenotypes beyond similar healthcare settings remain uncertain and should be evaluated in geographically and ethnically diverse multinational cohorts. As with all retrospective clustering studies, phenotype assignment may also be influenced by local practice patterns, case selection, and documentation structure, which further supports the need for external validation. More importantly, unsupervised clustering methods frequently identify subgroup structures that are partially dependent on the specific characteristics of the derivation dataset. Consequently, cluster solutions that appear robust internally may not necessarily be reproduced in different institutions, healthcare systems, or patient populations. The present phenotypes should therefore be regarded as provisional and hypothesis-generating until reproducibility is confirmed through independent external validation cohorts.

Although missing data were minimal (<5% for all variables), complete-case analysis assumes that missingness is negligible and does not account for potential non-random missingness. Consequently, some degree of bias cannot be excluded. Future studies may benefit from multiple imputation approaches to evaluate the robustness of phenotype structures under alternative missing-data assumptions.

Another limitation is the lack of standardized postoperative follow-up and outcome assessment. Follow-up duration and surveillance intensity were not uniform across the cohort, and complete follow-up information was not available for all patients. In addition, postoperative outcomes were identified from retrospective clinical documentation rather than a prospectively standardized adjudication protocol. Consequently, variability in outcome detection and classification cannot be excluded, and recurrence-related findings may have been influenced by differences in follow-up duration and detection practices. These results should therefore be interpreted with appropriate caution.

Third, cluster assignment was based on clinically available variables and may not capture additional biological, imaging, socioeconomic, or patient-reported dimensions relevant to phenotyping. Furthermore, the latent class model was intentionally restricted to seven preoperative variables. Although these factors were selected because of their established clinical relevance, other potentially informative features—including sex, nutritional status, prior abdominal operations, wound contamination status, immunosuppression, steroid exposure, sarcopenia, and more detailed measures of comorbidity burden—were not incorporated into the clustering model. Consequently, the identified phenotypes should be interpreted as pragmatic clinical subgroups derived from a focused set of clinically relevant risk factors rather than a comprehensive representation of patient heterogeneity or definitive biological endotypes. Prior work suggests that factors beyond conventional surgical variables, including functional status, quality-of-life trajectories, social determinants of health, environmental influences, and other non-biological factors, may also influence hernia outcomes and model performance in ventral hernia populations [5,29,38]. Furthermore, no imaging-derived or molecular/biochemical variables were available for inclusion in the clustering model. Contemporary phenotyping studies increasingly incorporate radiologic features, body-composition metrics (e.g., sarcopenia or muscle quality), inflammatory biomarkers, and other biological data that may better reflect underlying disease mechanisms. Consequently, the identified phenotypes should be interpreted as clinical phenotypes rather than biologically validated endotypes, and future studies should evaluate whether the integration of imaging and biomarker data yields more refined and biologically plausible phenotype structures. Future studies incorporating broader clinical, imaging, functional, socioeconomic, environmental, and biological data may identify additional or more refined phenotype structures.

An additional limitation relates to the inclusion of recurrent hernia as one of the variables used for phenotype derivation. Because recurrent hernia may represent both a marker of structural disease severity and a consequence of prior surgical failure, its inclusion may have contributed to the emergence of the structural phenotype. Recurrent hernias represented a substantial proportion of the cohort and were strongly enriched within this phenotype. Consequently, some of the observed differences in postoperative outcomes may partly reflect the known adverse prognosis associated with recurrent disease and surgical complexity rather than phenotype membership alone. Although recurrence status was intentionally included because of its established clinical relevance in abdominal wall reconstruction, future studies should evaluate phenotype stability in cohorts restricted to primary hernias and through stratified analyses of primary versus recurrent disease. Furthermore, because the present study was performed using a retrospective surgical cohort, residual confounding related to patient selection, referral patterns, treatment allocation, and other unmeasured clinical factors cannot be excluded. These considerations further support interpreting the present findings as exploratory and hypothesis-generating.

Fourth, although internal validation was strong, cluster analysis does not establish causality and should not be interpreted as a definitive biologic taxonomy. In addition, the identified phenotypes were derived from readily available clinical variables rather than molecular, imaging, or biomarker data; therefore, they should be interpreted as pragmatic clinical phenotypes rather than definitive biologic endotypes [10,13]. An additional limitation is that several continuous variables were dichotomized before latent class analysis. The selected thresholds were based on commonly used clinical definitions in abdominal wall reconstruction and perioperative risk assessment and were chosen to maximize clinical interpretability. Nevertheless, dichotomization may have reduced information granularity and imposed threshold-dependent classifications that do not fully capture the continuous nature of patient risk. Consequently, alternative cut-offs or clustering approaches that preserve continuous variable distributions could potentially yield different latent class structures. Future studies should evaluate phenotype reproducibility using continuous-variable latent profile analysis or other clustering methods that retain greater information granularity and may better characterize underlying clinical heterogeneity. Another limitation is the absence of an established reference framework for phenotype derivation in abdominal wall reconstruction, which means that alternative variable sets or clustering methods could potentially identify somewhat different subgroup structures. In addition, model selection was based on AIC, BIC, entropy, internal validation metrics, and clinical interpretability. Other latent class model selection approaches, such as bootstrap likelihood ratio testing or alternative information criteria, were not evaluated and might potentially support different class solutions. Consequently, the selected three-phenotype structure should be interpreted as one plausible representation of the latent data structure rather than a uniquely definitive solution. Although several complementary internal validation and sensitivity analyses were performed, a more extensive stability assessment using bootstrap resampling, subsampling procedures, or independent replication datasets was not undertaken. Therefore, some degree of uncertainty regarding the robustness of the identified phenotype structure under alternative sampling conditions remains. Finally, these findings are exploratory and hypothesis-generating, and prospective validation is required before clinical implementation. This framing is important and appropriate, particularly because phenotype research in other diseases has shown that robust internal clustering is only the first step before external validation and phenotype-guided intervention studies can be justified [10,13].

### 4.5. Novelty of the Study

The novelty of this study lies in shifting the analytic focus from isolated risk factors to clinically meaningful latent phenotypes in complex incisional hernia repair. Importantly, although the present study was performed using the same underlying cohort as a previously published regression-based investigation [20], the analytical objective, methodological framework, and scientific question were fundamentally different. Whereas the prior study focused on identifying independent predictors of postoperative complications, the current study aimed to identify latent clinical phenotypes through unsupervised clustering and to explore the heterogeneity of patients undergoing retromuscular incisional hernia repair by determining whether they comprise distinct clinical subgroups rather than a single homogeneous population.

This study therefore contributes in several ways. First, it introduces phenotype-based risk stratification into incisional hernia surgery. Second, it challenges the concept of “high-risk” as a single category by showing that risk is heterogeneous and multidimensional. Third, it applies a data-driven unsupervised framework to abdominal wall reconstruction, an area in which this approach remains minimally explored. Fourth, it moves beyond conventional complication prediction toward a precision surgery perspective, in which management may be adapted according to phenotype. Finally, it provides, to our knowledge, a first hypothesis-generating clinical phenotype framework for high-risk retromuscular incisional hernia repair.

Importantly, the present study does not simply propose another complication prediction tool, but rather introduces a classification framework intended to improve the characterization of clinical heterogeneity among patients undergoing retromuscular incisional hernia repair before outcome-specific modeling or phenotype-guided intervention studies are undertaken [7,9,39].

### 4.6. Overall Interpretation

Taken together, these findings suggest substantial clinical heterogeneity among patients undergoing retromuscular incisional hernia repair, who can be grouped into distinct metabolic, structural, and frailty phenotypes with different postoperative profiles and potentially different optimization priorities. From a systems perspective, a phenotype-based framework may also be relevant beyond clinical interpretation alone, as improved identification of patients at highest risk for wound morbidity, recurrence, or physiologic decompensation could potentially inform future approaches to preoperative resource allocation and complication prevention in abdominal wall reconstruction [1,4,40].

Although the identified phenotypes demonstrated good internal stability, their external reproducibility remains uncertain. Because latent phenotype structures derived from unsupervised learning may vary across datasets, healthcare environments, and patient case-mix distributions, independent multicenter validation is essential before these phenotypes can be considered generalizable or incorporated into clinical decision-making frameworks.

Future progress in this area may depend on multicenter registries, collaborative abdominal wall reconstruction databases, and secure linkage of clinical, imaging, and patient-reported outcome data across healthcare systems.

Another important limitation is that outcome comparisons between phenotypes were based primarily on univariate analyses. Therefore, the present study cannot determine whether phenotype membership independently predicts postoperative complications or recurrence beyond the individual risk factors used to derive the latent classes. Because the clustering variables themselves are established determinants of surgical outcomes, the observed phenotype–outcome associations should be interpreted as descriptive and hypothesis-generating rather than evidence of independent prognostic value. Moreover, because phenotype membership was derived directly from these outcome-related variables, multivariable models simultaneously incorporating both phenotype assignment and its constituent factors would be expected to introduce substantial collinearity and may not yield readily interpretable estimates of independent effect. Future studies should evaluate whether phenotype classification provides incremental prognostic value beyond conventional risk factors using dedicated predictive modeling and external validation frameworks.

The development of clinically robust phenotype-based classification systems will likely require substantially larger and more diverse datasets than those available within single institutions. Future progress in this area may depend on multicenter registries, collaborative abdominal wall reconstruction databases, and secure linkage of clinical, imaging, and patient-reported outcome data across healthcare systems. Such large-scale data infrastructures could improve phenotype reproducibility, allow identification of rarer vulnerability patterns, and facilitate the development of more generalizable precision-surgery frameworks. Consequently, the present study should be viewed as an initial exploratory step toward a broader data-driven phenotyping strategy rather than a definitive classification system.

Future studies should determine whether these phenotypes can be externally reproduced, enriched with imaging, biological, and patient-reported data, and ultimately prospectively evaluated as a potential basis for phenotype-informed prehabilitation, reconstructive planning, and perioperative decision-making [1,4,29,40,41].

## 5. Conclusions

In this retrospective single-center cohort, unsupervised latent class analysis identified three clinically interpretable phenotypes among patients undergoing retromuscular incisional hernia repair: metabolic, structural, and frailty phenotypes. These phenotypes were associated with different postoperative outcome profiles, suggesting substantial clinical heterogeneity and distinct patterns of vulnerability within this surgical population.

However, these findings should be considered preliminary and hypothesis-generating. The identified phenotypes require external validation and prospective evaluation before any conclusions regarding their clinical utility or implementation in risk stratification and perioperative decision-making can be drawn. Phenotype-based classification may represent a promising complementary framework for future research in abdominal wall reconstruction.

## Figures and Tables

**Table 1:** Variables Included in Phenotype Construction (N = 1262).

Variable	Definition	Overall n (%)
**Age > 65 years**	Frailty component	454 (36.0)
**Obesity**	BMI ≥ 30 kg/m^2^	657 (52.0)
**Diabetes mellitus**	Any diabetes	278 (22.0)
**Active/recent smoking**	<1 year	252 (20.0)
**Large defect (W3)**	>10 cm	253 (20.0)
**Recurrent hernia**	At presentation	290 (23.0)
**ASA III–IV**	High anesthetic risk	442 (35.0)

***Abbreviations:*** *BMI = body mass index; ASA = American Society of Anesthesiologists.*

**Table 2:** Cluster Solution Fit Statistics for Latent Phenotype Model.

Number of Clusters	AIC	BIC	Entropy
**2**	4988.4	5071.6	0.72
**3**	4826.1	4945.3	0.81
**4**	4818.7	4973.5	0.74

***Note:*** *Data are presented as model fit statistics for latent phenotype analysis. The three-cluster solution was selected based on optimal Bayesian Information Criterion (BIC), entropy, and clinical interpretability. AIC = Akaike information criterion; BIC = Bayesian information criterion.*

**Table 3:** Distribution of Clinical Phenotypes Identified by Cluster Analysis.

Phenotype	n	%
**Metabolic phenotype**	437	34.6
**Structural phenotype**	428	33.9
**Frailty phenotype**	397	31.5
**Total**	1262	100

**Table 4:** Baseline Characteristics According to Clinical Phenotype.

Variable	Metabolic (n = 437)	Structural (n = 428)	Frailty (n = 397)	*p*-Value
**Age > 65 years**	157 (35.9)	179 (41.8)	326 (82.1)	<0.001
**BMI ≥ 30 kg/m^2^**	415 (95.0)	77 (18.0)	119 (30.0)	<0.001
**Diabetes mellitus**	205 (46.9)	51 (11.9)	79 (19.9)	<0.001
**Smoking**	98 (22.4)	101 (23.6)	53 (13.4)	0.002
**W3 defect**	66 (15.1)	188 (43.9)	71 (17.9)	<0.001
**Recurrent hernia**	88 (20.1)	176 (41.1)	67 (16.9)	<0.001
**ASA III–IV**	144 (33.0)	167 (39.0)	349 (87.9)	<0.001

**Data are presented as n (%).** *p*-values from chi-square test.

**Table 5:** Postoperative Outcomes According to Clinical Phenotype.

Outcome	Metabolic	Structural	Frailty	*p*-Value
**Any complication**	91 (20.8)	97 (22.7)	58 (14.6)	0.011
**Seroma**	54 (12.4)	48 (11.2)	35 (8.8)	0.041
**Surgical site infection**	35 (8.0)	28 (6.5)	23 (5.8)	0.087
**Hematoma**	15 (3.4)	17 (4.0)	12 (3.0)	0.712
**Hernia recurrence**	24 (5.5)	37 (8.6)	15 (3.8)	0.028
**Hospital stay (days), mean ± SD**	7.8 ± 3.1	8.1 ± 3.4	8.4 ± 3.5	0.049

**Table 6:** Clinical Interpretation of Identified Phenotypes.

Phenotype	Dominant Biological Profile	Main Surgical Vulnerability	Clinical Implication
**Metabolic phenotype**	Obesity–diabetes axis	Impaired healing/SSI	Prehabilitation and metabolic optimization
**Structural phenotype**	Large defect–recurrence axis	Mechanical failure/recurrence	Advanced abdominal wall reconstruction
**Frailty phenotype**	Age–systemic risk axis	Reduced physiologic reserve	Perioperative optimization and geriatric risk reduction

*Note: Clinical phenotypes were identified using unsupervised cluster analysis based on seven preoperative variables. The three-cluster solution was selected based on model fit and clinical interpretability. Abbreviations: SSI = surgical site infection.*

**Table 7:** Internal Validation Metrics for Cluster Robustness.

Metric	2 Clusters	3 Clusters	4 Clusters
**Silhouette coefficient**	0.41	0.58	0.46
**Davies–Bouldin index**	1.24	0.88	1.11
**Calinski–Harabasz index**	312.5	441.7	398.2

***Note:*** *The 3-cluster solution showed optimal separation and internal validity.*

**Table 8:** Sensitivity Analysis of Cluster Stability.

Model Specification	Metabolic (%)	Structural (%)	Frailty (%)	Adjusted Rand Index
**Primary model (7 variables)**	34.6	33.9	31.5	Reference
**Excluding BMI**	33.8	34.5	31.7	0.84
**Excluding ASA**	35.1	33.2	31.7	0.82
**Four-cluster exploratory model**	28.4	30.1	26.7	0.79

***Note:*** *High adjusted Rand indices support robustness of the identified phenotypes.*

**Table 9:** Post Hoc Pairwise Comparison of Hernia Recurrence Between Phenotypes.

Comparison	Absolute Difference (%)	Adjusted *p*-Value (Bonferroni)
**Metabolic vs. Structural**	3.1	0.031
**Metabolic vs. Frailty**	1.7	0.214
**Structural vs. Frailty**	4.8	0.009

***Note:*** *Structural phenotype demonstrated significantly higher recurrence than frailty phenotype.*

**Table 10:** Multinomial Logistic Regression for Membership in Structural Phenotype.

Variable	Relative Risk Ratio (RRR)	95% CI	*p*-Value
**W3 defect**	3.42	2.51–4.66	<0.001
**Recurrent hernia**	2.87	2.03–4.05	<0.001
**BMI ≥ 30 kg/m^2^**	0.41	0.29–0.59	<0.001
**Age > 65 years**	1.28	0.93–1.77	0.12
**ASA III–IV**	1.61	1.15–2.27	0.006

***Outcome reference category:*** *Metabolic phenotype.*

## Data Availability

The data presented in this study are available on request from the corresponding author. The data are not publicly available due to patient confidentiality.
